# Long-term quality of life after decompressive craniectomy

**DOI:** 10.3389/fneur.2023.1222080

**Published:** 2023-07-26

**Authors:** Daniel Buffagni, Alvaro Zamarron, Isabel Melgosa, Raquel Gutierrez-Gonzalez

**Affiliations:** ^1^Department of Surgery, Faculty of Medicine, Autonomous University of Madrid, Madrid, Spain; ^2^Department of Neurosurgery, Puerta de Hierro University Hospital, IDIPHISA, Madrid, Spain; ^3^Department of Anesthesiology, Marques de Valdecilla University Hospital, Santander, Spain

**Keywords:** brain injuries, traumatic, decompressive craniectomy, intracranial hypertension, morbidity, quality of life

## Abstract

**Introduction:**

This study aims to assess the quality of life (QoL) in patients who have undergone decompressive craniectomy (DC) for any pathology that has caused life-threatening intracranial hypertension. Similarly, it aims to evaluate QoL perceived by caregivers or external informants. In addition to that, the last purpose is to determine which clinical or therapeutic factors could correlate with a better QoL.

**Methods:**

A single-center cross-sectional study was designed. All patients over 18 years old who underwent a supratentorial DC at our department due to intracranial hypertension of any etiology, from January 2015 to December 2021, were retrospectively selected. Patients with incomplete follow-up (under 1 year from the event or those who died) or who declined to participate in the study were excluded. QoL was assessed with SF-36 and CAVIDACE scales. The correlation between clinical and therapeutic variables and SF-36 subscales was studied with Spearman's correlation and the Mann–Whitney U-test.

**Results:**

A total of 55 consecutive patients were recruited: 22 patients had died, three were missed for follow-up, and 15 declined to participate, thus 15 subjects were finally included. The mean follow-up was 47 months (IQR 21.5–67.5). A significant reduction in the “role physical” and “role emotional” subscales of SF-36 was observed compared with the general population. According to caregivers, a significant reduction was assigned to the “physical wellbeing” and “rights” domains. The “physical functioning” score was poorer in women, older patients, those with dominant hemisphere disease, those who required tracheostomy, and those with poor outcomes in the modified Rankin scale. A strong correlation was found between the QoL index at the CAVIDACE scale and the SF-36 subscales “physical functioning” and “role physical”.

**Conclusion:**

Most patients and caregivers reported acceptable QoL after DC due to a life-threatening disease. A significant reduction in SF- 36 subscales scores “role limitation due to physical problems” and “role limitation due to emotional problems” was referred by patients. According to caregivers' QoL perception, only 25% of the survey's participants showed low scores in the QoL index of the CAVIDACE scale. Only 26.7% of the patients showed mood disorders.

## 1. Introduction

The skull is a rigid, non-expandable compartment, therefore increased intracranial volume may lead to uncontrolled intracranial hypertension with subsequent cerebral ischemia and tissue death. The most common cause of this condition is traumatic brain injury (TBI) and stroke. However, it can also occur in the context of hydrocephalus, tumors, infections, hemorrhage, and certain encephalopathies. At the beginning of the 20th century, neurosurgeons such as Kocher and Cushing systematically described techniques for removing cranial bone flaps to treat pathologies that caused an increase in intracranial pressure (1). Thus, decompressive craniectomy (DC) was born. The technique consists of removing a region of the skull to allow the expansion of the cranial content subjected to volume expansion processes. Over the years, this technique has not been free of skepticism. Although it has been described as a valuable technique for reducing mortality in pathologies, such as severe TBI or malignant middle cerebral artery (MCA) infarction, it is not clear whether patients who survive show a higher degree of disability (2–5). They also require a second surgical procedure to replace the bone flap several weeks or months later whenever the clinical condition improves and allows it.

Few studies have assessed the patient's quality of life (QoL) among those who survived after this procedure. Although QoL is challenging to determine, it provides a more humanistic and holistic approach to medicine, focusing less on clinical aspects and taking more into account the patient outside the healthcare environment. It is interesting to know how this procedure influences QoL, especially to analyze health-related QoL (HRQoL), which assesses the physical, psychological, social, and functional situations. Different scales, such as the Medical Outcomes Study 36-item Short-Form Health Survey (SF-36) (6, 7), the EuroQoL (8, 9), the “Sickness Impact Profile” (SIP) (10), different versions of the “Stroke Impact Scale” (SIS) (11, 12), or the “Quality of Life after Brain Injury” scale (QOLIBRI) (13, 14), have been used for this purpose.

Most studies focused on MCA infarction. A systematic review concluded that QoL was acceptable and that patients were satisfied with the treatment. It also showed that severe depression was rare (<16%), but mild depressive symptoms were quite common (48.5%). Finally, it also considered and studied the caregiver burden, identifying high levels of stress in 70% of caregivers (15). Another review concluded that QoL was reduced in many cases and showed better functional outcomes after DC in younger patients and those with fewer co-morbid conditions (16). Other TBI studies also concluded that QoL among survivors was moderate or good (3, 13, 14). Kelly et al. compared patients with severe TBI who underwent DC with those who underwent craniotomy (bone replacement in the same procedure). They observed similar results in life scale scores, but they also identified more extended hospital stays, decreased functional status, and more rehospitalizations in patients undergoing hemicraniectomy (17). A recent study on spontaneous intracerebral hemorrhage, analyzing 290 patients, also compared craniotomy with DC. The authors observed higher scores in GOS and SF-36 scales at 6-month follow-up in patients undergoing DC (7). Finally, D'Ambrosio et al. evaluated 22 patients with poor-grade aneurysmal subarachnoid hemorrhage, observing poor QoL in DC survivors (8).

In general conclusion, DC offers patients an acceptable QoL (3, 6, 14), and in any case, it is better than if it had not been used since most non-surgical patients would eventually die. However, studies published in our country are yet to analyze this perspective. Only one study has been published in this regard. Benejam et al. assessed QoL in 19 survivors of MCA infarction. They found a higher reduction in the SIP's physical domain than in the psychosocial domain and registered depressive symptoms in 50% of patients. However, the outcome assessment was performed 7.47 months after the infarction in an active recovery phase (10).

This study aims to assess the QoL in patients who have undergone DC for any pathology that has caused life-threatening intracranial hypertension. Similarly, it aims to evaluate QoL perceived by caregivers or external informants. The last purpose is to determine which clinical or therapeutic factors could correlate with a better QoL.

## 2. Materials and methods

A single-center cross-sectional study was designed to assess the QoL in patients undergoing DC. The study was approved by the local Ethics Committee of Puerta de Hierro University Hospital (reference 195/22) and was conducted under the 1964 Helsinki Declaration and its later amendments or comparable ethical standards.

### 2.1. Patients' selection

All consecutive patients over 18 years old who attended and underwent a supratentorial DC at our department due to intracranial hypertension of any etiology from 1 January 2015 to 31 December 2021 were retrospectively selected for analysis. Patients with incomplete follow-up (under 1 year from the event or those who died) or who declined to participate in the study were excluded. Informed consent was obtained from all participants before any intervention.

### 2.2. Outcome variable

QoL was assessed employing two tools. Patients completed the MOS 36-Item Short-Form Health Survey (SF-36) validated in Spanish (18), whereas caregivers or cohabitants filled out the CAVIDACE scale (19). SF-36 is an instrument used transversally to assess QoL in the general population and in patients suffering from different pathologies. It is considered a good indicator of HRQoL. The patient answers 36 questions covering eight main domains or subscales: physical functioning, role physical, bodily pain, social functioning, mental health, role emotional, vitality, and general health. Scores range from 0 to 100, and higher scores involve better QoL. The results were compared with a national population-based study (20).

The CAVIDACE scale is elaborated in Spanish and validated in the Spanish population with acquired brain injury. It is used to assess adult patients with brain damage, defined as those over 18 years of age outside the school environment. It is completed by an external informant who has known the subject for at least 3 months. Eight dimensions of QoL are assessed: emotional wellbeing, material wellbeing, interpersonal relationships, physical wellbeing, rights, personal development, self-determination, and social inclusion. Each domain is evaluated with eight Likert scale questions rated from 0 to 3, where 0 means never and 3 means always. Each domain is given a direct score that correlates with a standardized score (0–18) and the associated percentile (<1–99). Then, a QoL index can be calculated and associated with a QoL percentile.

### 2.3. Independent variables

Epidemiological, clinical, diagnostic, and therapeutic variables were registered and included in the dataset to ascertain any association with QoL. These variables included age, gender, medical and surgical history, the diagnosis that led to DC, dominant hemisphere DC, the interval from admission to DC, Glasgow Coma Scale (GCS) score at the moment of DC decision, the need for tracheostomy, external ventricular drain or ventriculoperitoneal shunt, intensive care unit (ICU) stay, and hospital stay. This information was retrospectively obtained from the electronic health record. Moreover, other information was collected prospectively during an interview with the patient and the accompanying person: rehabilitation therapy duration, functional outcome assessed by the modified Rankin scale (mRS) (21), mood disorder diagnosis from a clinician along all the follow-up, and social support.

### 2.4. Statistical analysis

Dataset information was processed and analyzed using StataCorp. 2019 (Stata Statistical Software: Release 16. College Station, TX: StataCorp LLC). Numerical normally distributed variables were represented by the mean and standard deviation (SD) whereas not normally distributed ones were described by the median and 25th and 75th percentiles. Absolute and relative frequencies were used in categorical variables and as the description measure. Z-scores were calculated to compare the results on SF-36 obtained in the sample with the general population in our country (20) with Spearman's rank correlation coefficient. A difference between the sample mean and the population mean equal to or greater than one SD was considered clinically relevant.

Non-parametric tests were used to analyze any correlation between SF-36 subscales and different epidemiological, clinical, diagnostic, and therapeutic variables due to the reduced sample size. Thus, Spearman's correlation test for continuous variables and the Mann–Whitney U-test for categorical variables were used. Every statistical hypothesis was two-tail tested. The null hypotheses with type I error or α error of <0.05 were rejected in all hypothesis contrasts.

## 3. Results

**Figure 1:**
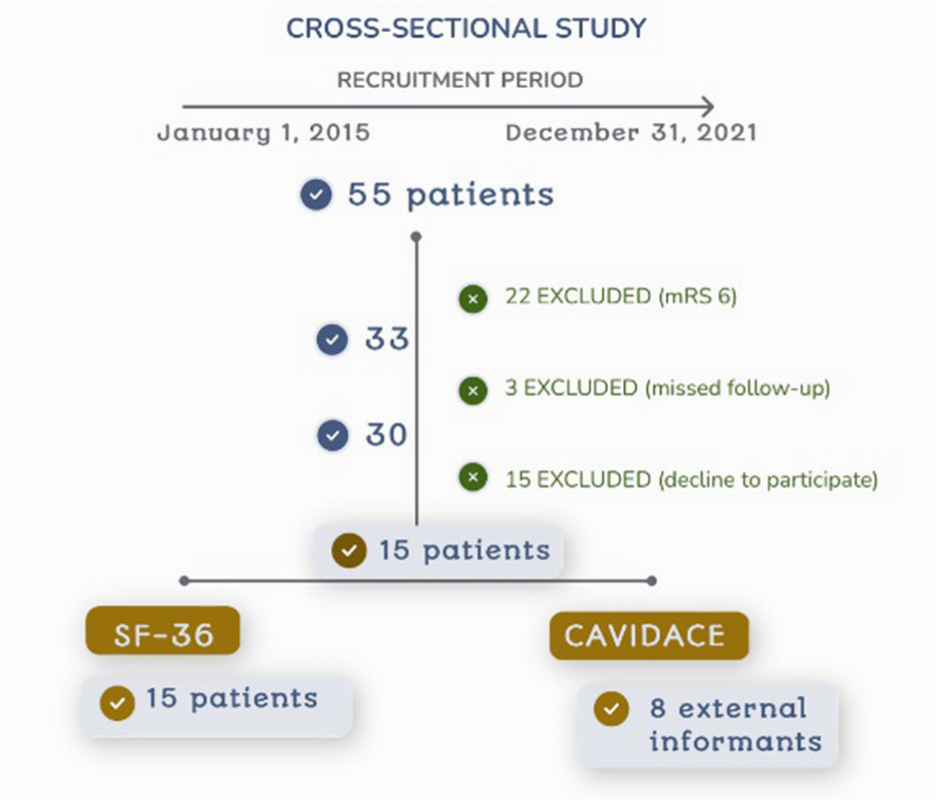
Flowchart of the recruitment process.

A total of 55 consecutive patients were initially included. The incomplete follow-up excluded 22 patients who died after the procedure (40%), 3 missed follow-up, 15 patients declined to participate, thus 15 subjects were finally included in the study and signed the informed consent (Figure 1).

Male prevalence was shown in the sample (66.7%), and the mean age at diagnosis was 42.2 years (SD 17). All patients showed an mRS of 0 before symptom onset. The most frequent diagnosis that led to DC was TBI (60%), followed by meningoencephalitis (13.3%). One patient (6.7%) suffered a spontaneous intraparenchymal hemorrhage; another suffered an aneurysmal subarachnoid hemorrhage; and another underwent DC due to sinus thrombosis with subarachnoid hemorrhage. A complication (vascular rupture) after endovascular treatment of an MCA infarction was the reason for performing DC in the last case. The dominant hemisphere was decompressed in 40% of patients. According to the GCS score at the moment of DC decision, two patients (13.3%) showed scores of 14–15, five patients (33.3%) showed scores of 9–13, and seven patients (46.7%) presented with a score of 8 or less (6 of them had GCS score of 3 or 4). Table 1 summarizes patients' characteristics.

**Table 1:** Epidemiological, clinical, diagnostic, and therapeutic characteristics of the cohort.

**Variables**	**Cohort (*n* = 15)**
Male gender	10 (66.7%)
Mean age (SD), yr	42.2 (10.7)
mRS before onset (IQR)	0 (0; 0)
Diagnosis	
TBI	9 (60.0%)
Infection	2 (13.3%)
SAH	1 (6.7%)
Intracerebral hemorrhage	1 (6.7%)
Other	2 (13.3%)
Dominant hemisphere	6 (40.0%)
Median GCS at surgery (IQR)	9 (3; 11.5)
Median interval from onset to DC (IQR), h	12 (5.5; 72)
Tracheostomy	8 (53.3%)
EVD	2 (13.3%)
VP shunt	3 (20.0%)
Median ICU stay (IQR), dy	16 (11; 23)
Median hospital stay (IQR), dy	36 (32; 53)
Median rehab therapy (IQR) dy	180 (65; 363)
Social support	10 (66.7%)
Mood disorder	4 (26.7%)
mRankin score at interview (IQR)	1 (0.5; 2)

SD, standard deviation; mRS, modified Rankin score; IQR, interquartile range; TBI, traumatic brain injury; SAH, subarachnoid hemorrhage; GCS, Glasgow Coma Scale; DC, decompressive craniectomy; EVD, external ventricular drainage; VP, ventriculoperitoneal; ICU, intensive care unit.

The median time from symptom onset to DC was 12 h (5.5; 72), and most patients (66.7%) underwent an early decompression (during the first 24 h of symptoms). The median ICU stay was 16 days (11; 23), and the median hospital stay was 36 days (32; 53). The median time to cranioplasty was 100 days (52.5; 225). Most patients (66.7%) completed at least 3 months of rehabilitation therapy after the event, and most of them (66.7% of all patients) received social support. Only 26.7% of patients suffered from a mood disorder at any point in the evolution of their process (two patients presented depression, and two patients suffered mixed anxiety-depressive disorder; all of them scored <4 in mRS). The functional outcome was poor (mRS of 4-5) in two patients (13.3%), middle (mRS of 2–3) in five patients (33.3%), and good (mRS of 0–1) in the remaining eight patients (53.3%) at the interview.

The median follow-up was 47 months (21.5; 67.5). The mean age of patients at the moment of accomplishing the survey was 46.4 years (SD 16.8). All patients filled out the SF-36 questionnaire, but only eight external informants completed the CAVIDACE scale (53.3%). Scores on the SF-36 subscales are shown in Table 2. Considering self-perceived QoL, a significant reduction was obtained in subscales “role physical” and “role emotional” compared with the general population. In addition, a non-significant reduction in “physical functioning” and “social functioning” was also observed, with *z-scores* −0.79 and −0.71, respectively.

**Table 2:** SF-36 questionnaire results.

**Subscale**	**Sample mean (SD)**	**Population mean (SD)**	***Z*-Score**
Physical functioning	65.7 (34.5)	84.7 (24.0)	−0.79
Role physical	46.7 (47.1)	83.2 (35.2)	−1.04
Bodily pain	71.3 (30.3)	79.0 (27.9)	−0.28
General health	55.3 (25.1)	68.3 (22.3)	−0.58
Vitality	66.7 (23.0)	66.9 (22.1)	−0.01
Social functioning	75.9 (21.4)	90.1 (20.0)	−0.71
Role emotional	51.1 (45,2)	88.6 (30.1)	−1.25
Mental health	71.5 (16.6)	73.3 (20.1)	−0.09

SD, standard deviation.

According to patient caregivers' QoL perception, 75% of the surveys (6/8) presented QoL index-related percentiles between p61 and p98. In contrast, the remaining 25% (2/8) showed lower scores (QoL index-related percentiles p3 and p59). Percentiles on CAVIDACE domains are shown in Table 3. A significant reduction was obtained in the domains of “physical wellbeing” and “rights” (values equal to or under p50).

**Table 3:** CAVIDACE scale results and percentiles.

**Domain**	**Sample mean (SD)**	**Percentile**
Physical wellbeing	14.6 (5.88)	**p25**
Material wellbeing	20.1 (6.49)	p63
Personal development	17.0 (5.5)	p84
Rights	18.5 (4.44)	**p50**
Self-determination	17.9 (5.03)	p75
Social inclusion	17.4 (8.33)	p75
Interpersonal relationships	16.8 (5.23)	p75
Emotional wellbeing	18.1 (4.05)	p75
**QoL INDEX**	109 (17.1)	p72

SD, standard deviation; QoL, quality of life.

Bold: values equal to or under p50.

Finally, the analysis of the correlation between SF-36 subscales and different variables is summarized in Table 4.

**Table 4:** Correlation between SF-36 subscales' score and patient's variables, expressed in *p*-values.

**Variable**	**Physical functioning**	**Role physical**	**Bodily pain**	**General health**	**Vitality**	**Social functioning**	**Role emotional**	**Mental health**
Gender	**0.031**	0.262	0.121	0.296	0.218	0.754	0.846	0.579
Age (diagnosis)	**0.039**	0.170	0.579	0.090	0.575	0.202	0.177	0.331
TBI	0.440	1.000	0.633	1.000	0.722	1.000	0.803	0.859
Dominant hemisphere	**0.002**	0.076	0.233	0.236	0.594	0.809	0.950	0.476
GCS <9	0.726	0.319	0.860	0.485	0.727	0.679	0.807	0.954
Early DC	0.497	0.114	0.664	0.176	1.000	0.661	0.365	0.711
Tracheostomy	**0.020**	0.105	0.639	0.771	1.000	0.344	0.463	0.816
EVD	0.266	0.082	0.863	0.865	0.798	0.862	1.000	0.494
VP shunt	0.216	0.938	1.000	0.612	0.828	0.605	0.321	0.611
ICU stay	0.779	0.730	**0.047**	0.238	0.112	**0.026**	0.248	0.701
Hospital stay	0.878	0.591	0.317	0.086	0.975	0.941	0.424	0.107
Mood disorder	0.292	0.527	0.209	0.646	0.076	0.640	0.408	1.000
Social support	0.621	0.843	0.951	0.580	0.498	0.661	0.846	0.423
Poor mRS	**0.049**	0.522	0.491	0.932	0.172	0.297	0.590	0.669
Rehab > 3 mth	0.073	0.692	0.121	**0.027**	0.176	0.661	1.000	0.217
Follow-up	0.779	**0.028**	0.477	0.556	0.757	0.144	0.593	0.759
CAVIDACE QoL index (percentile)	**0.009**	**<0.001**	0.220	0.213	0.165	0.839	0.652	**0.051**

Bold: *p*-value <0.05.

**Figure 2:**
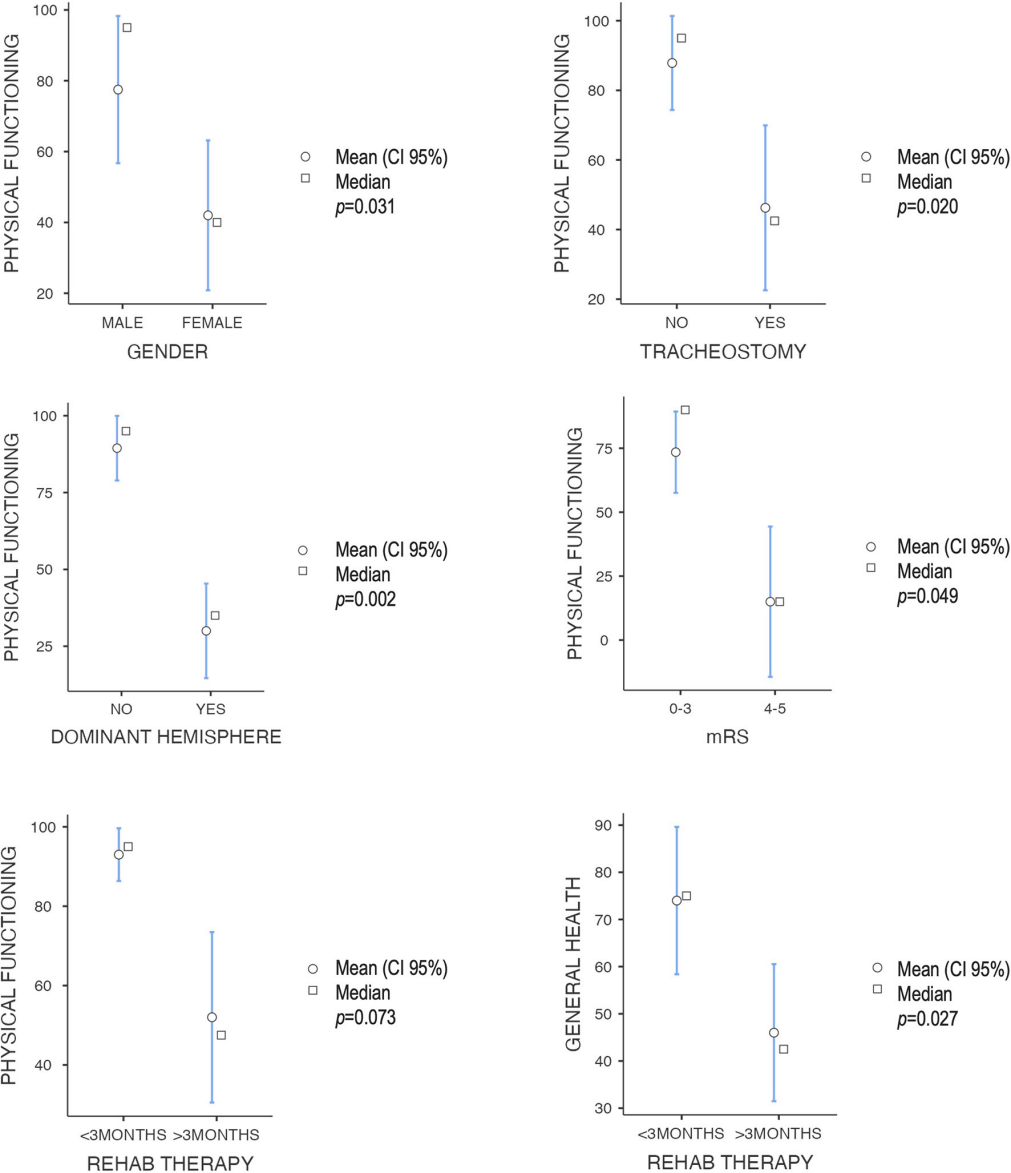
Correlation between different dichotomic clinical-therapeutic factors and SF-36 subscales, all with a *p* < 0.05 except extended rehabilitation therapy—physical functioning (Mann–Whitney *U*-test).

**Figure 3:**
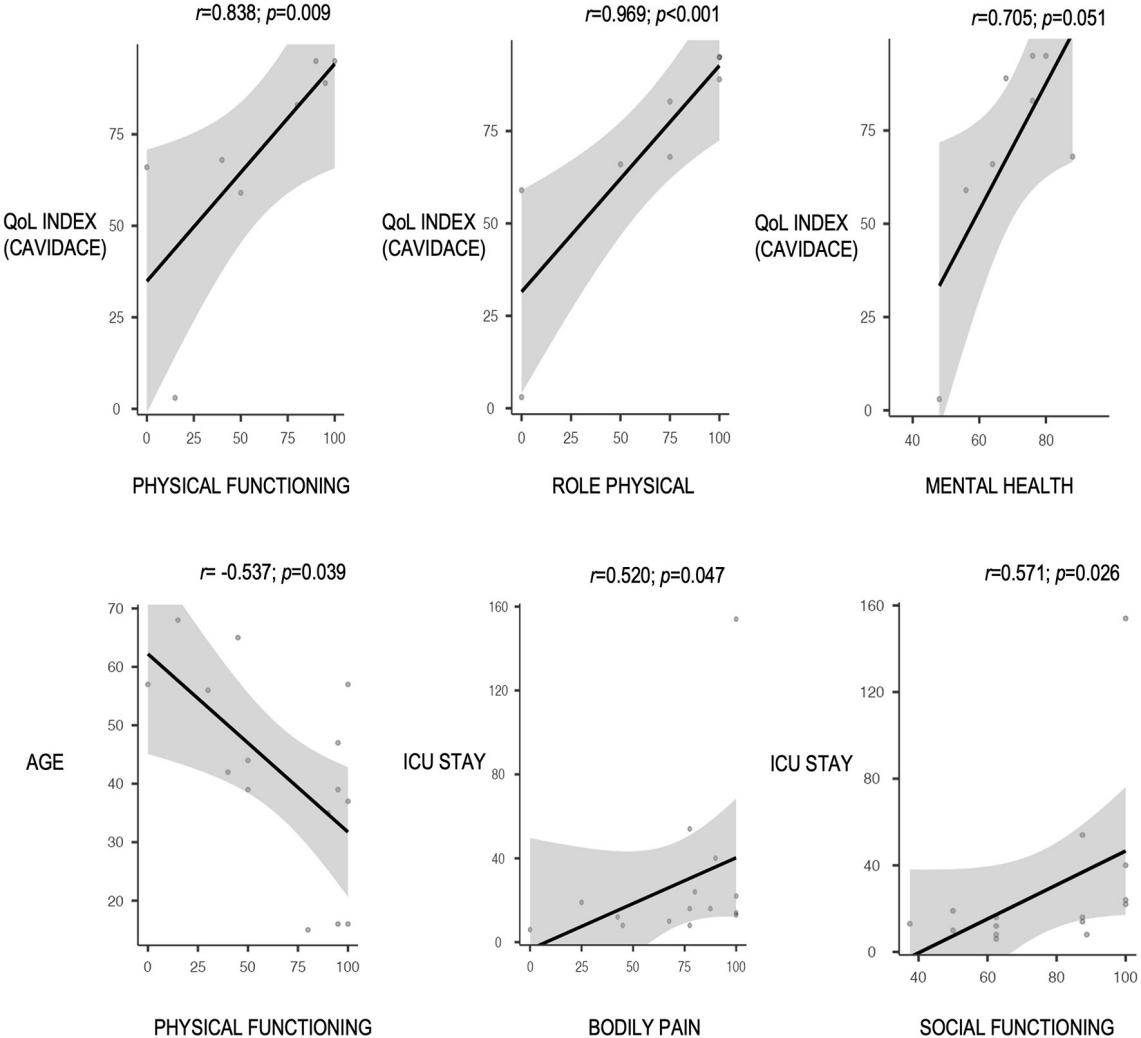
Correlation between different continuous clinical-therapeutic factors and SF-36 subscales, all with a *p* < 0.05 (Spearman's correlation test).

The “physical functioning” score was poorer in women, older patients, those with dominant hemisphere disease, those who required tracheostomy, and those with poor outcomes in the modified Rankin scale (Figure 2). A positive correlation was observed between the QoL index percentile at the CAVIDACE scale and the subscales “physical functioning”, “role physical” and “mental health” (Figure 3). In the latter case, the statistical significance was marginal (*p* = 0.051). Positive correlations were also confirmed between the follow-up and “role physical”; length of ICU stay and “bodily pain” or “social functioning” (Figure 3); the correlation was negative between rehab therapy over three months and “general health”. An almost significant negative correlation was also detected between extended rehab therapy and “physical functioning” (Figure 2). Finally, no correlation was evidenced between the analyzed variables and the domain “role emotional”.

## 4. Discussion

This research focuses on the QoL perceived by the patient and the caregivers at least 1 year after undergoing an emergent DC due to a life-threatening disease. A significant reduction of expected values in one physical (role limitation due to physical problems) and one mental domain (role limitation due to emotional problems) was observed. The remaining subscale scores were comparable to those of the general population. According to external informants' evaluation, the lowest scores were assigned to “physical wellbeing” and “rights”. A strong correlation was found between the QoL index at the CAVIDACE scale and the SF-36 subscales “physical functioning” and “role physical”. This association showed borderline significance with the domain “mental health”.

The technique of DC has been associated with increased survival in cases of intracranial hypertension but with a more disabling functional outcome (2). However, the QoL that patients perceive is not so far from the general population (3). Research published is not homogeneous since it includes different diagnoses and tools to assess the outcome. Similarly, most studies include small sample sizes. The SF-36 questionnaire was selected for this project since it is easy to perform, it is world-wide accepted, and national population data are available for comparison. This questionnaire's subscale “role physical” refers to limitations in usual role activities because of physical health problems. Then, motor impairments may influence this result. Several studies on stroke have observed that the physical summary is altered (6, 9, 10, 12, 22–24). We also determined lower scores in physical subscales compared with the general population, but no patient with MCA infarction was enrolled in this study. However, when analyzing other pathologies, such as spontaneous hemorrhage or TBI, the results showed better physical component outcomes than those obtained in patients undergoing craniotomy but in the absence of comparison with the general population (7, 14).

The “role emotional” domain of the SF-36 questionnaire deals with the ability to perform work and other activities depending on the presence of emotional problems, whereas “social functioning” refers to the interference that emotional problems may cause in the patient's normal social activities. Both are used to measure the psycho-psychiatric involvement associated with DC without establishing a clinical diagnosis of depression or anxiety. This is complemented by the results obtained with the “mental health” item, which focuses on the development of depression and anxiety. In our experience, DC was associated with an emotional impairment represented by altering the “role emotional” but without causing a significant mood or adaptive disorder, keeping “mental health” within values comparable to the general population.

Depressive and anxious symptoms were present in 26.7% of the patients in this study, where no difference was obtained in the “mental health” subscale. This fact may be related to the diagnosis that led to DC since post-stroke depression appears in 18–55% of cases (10, 23, 25), and no patient with that diagnosis participated in our study. Other studies have found an association between depression and the use of DC in ischemic stroke. In this regard, some authors have described no correlation with a physical disability (26), whereas others have associated the severity of anxiety or depression with the severity of disability (27).

It is important to consider the time from DC to the completion of the survey since several studies report short-term QoL (7, 10, 27). Neurological recovery after a life-threatening event is faster initially but may last for more than 1 year, so early evaluation may not represent health for the rest of their lives (9). We only included patients with a minimum follow-up of 1 year; the median time elapsed from the event to the outcome assessment was almost 4 years (47 months; IQR 21.5–67.5). QoL was considered poor at a 1-year follow-up after poor-grade aneurysmal subarachnoid hemorrhage (8) but reasonable in MCA infarction patients after a mean follow-up of 8 years (9) or even good after TBI up to 10 years after injury (14). Then, it is essential to consider long-term outcomes in decision-making.

Caregivers' perception of QoL has been reported in a few studies. In most cases, they would decide to perform DC again (6, 10, 13, 16, 23, 28), with one exception where half of the patients would decide against it (27). We observed that only 25% of the survey's participants referred to low scores in the QoL index of the CAVIDACE scale. Even though there is no significant association between all SF-36 subscales and the QoL index of the CAVIDACE scale, it is interesting to highlight the strong correlation between the index and the subscales “physical functioning” and “role limitation due to physical problems,” as well as the borderline association with the domain “social functioning.” This finding could be applicable in further studies where patients unable to respond to a questionnaire could be evaluated with the participation of their relatives or caregivers with an adequate estimation of their perceived QoL, which is ultimately the most relevant.

Several studies have determined an association between better functional status and younger patients (10, 16, 26, 27). Other authors have also observed higher QoL in younger patients (10). Only Rauen et al. obtained better scores in HRQoL in older patients (14). Nevertheless, no study has detected a correlation between QoL and sex. This study observed that women and older patients had significantly impaired physical QoL. However, women were older (mean age 53) than men (43.1 years old), so a multivariable analysis should be performed to detect confounding factors. No association was confirmed between the initial GCS score or the elapsed time between life-threatening injury and DC, as identified by other studies (8, 10). However, the need for tracheostomy also correlated with poorer results in the “physical functioning” subscale. This feature is usually related to more extended ICU stays and has been associated with worse disability (27). In this case, a prolonged ICU stay positively correlated with “bodily pain” and “social functioning” domains, a finding difficult to interpret. Then, further studies with larger sample sizes are needed to confirm these results since no study has analyzed these variables to date.

The injury of the dominant hemisphere has also been assessed in several studies, but no significant difference in QoL has been observed compared to non-dominant damage (6, 10, 15, 28, 29). In our experience, dominant hemisphere injury significantly correlated with poorer “physical functioning” scores. This finding must be explained since language impairment hampers rehabilitation activities. Moreover, the affectation of the dominant motor area makes it more complicated to carry out physical activities since the dominant arm and leg perform most of them more precisely, relying on the action of the contralateral limb. In this same line of argumentation, poor functional outcomes (mRS score of 4–5) showed the same association with worse “physical functioning” scores. On the contrary, Benejam et al. reported that QoL was unrelated to mRS scores (10). Finally, an extended rehabilitation program over 3 months showed a significant correlation with lower scores in the “general health” domain and an almost significant association with lower values in the “physical functioning” subscale of the SF-36. This finding may be related to the functional outcome since more prolonged therapies are usually needed when the patient shows a worse disability. In contrast, those patients with better functional status do not require therapy at discharge. This conclusion is yet to be reported, so no comparisons can be made. Finally, two out of three patients showed social support, but no correlation could be established between this variable and QoL.

Several limitations must be outlined. The first one arises from the sample size although significant results have been obtained. Moreover, several studies published to date share this feature. This may explain the discrepancy between our results and those of other more robust studies. Then, further studies recruiting more patients and including multivariable analysis to detect confounding factors are needed. Second, the quality of the scales currently used to assess HRQoL must be considered as another limitation. On the one hand, the study in the general population that serves as a reference for comparison dates from 1998 (25 years ago). On the other hand, using different non-homogeneous scales hinder the comparability of other studies. Then, the interpretation of the data must be cautious. Since this is a cross-sectional study, a causal order of the correlations observed cannot be established. Finally, no patients with MCA infarction participated in the study. Since most of the literature related to QoL and DC is studied in these patients, comparison of the results is difficult.

## 5. Conclusion

A significant reduction in SF-36 subscales “role limitation due to physical problems” and “role limitation due to emotional problems” was observed in self-perceived QoL compared with the general population. According to patient caregivers' QoL perception, only 25% of the survey's participants showed low scores in the QoL index of the CAVIDACE scale. The lowest scores were obtained in “physical wellbeing” and “rights”. Only 26.7% of the patients showed mood disorders.

The “physical functioning” score was poorer in women, older patients, those with dominant hemisphere disease, those who required tracheostomy, and those with poor outcomes, according to the mRS. A strong correlation was found between the QoL index at the CAVIDACE scale and the SF-36 subscales “physical functioning” and “role physical”, as well as a borderline correlation with the “mental health” domain.

## Data availability statement

The datasets presented in this study can be found in online repositories. The names of the repository/repositories and accession number(s) can be found at: Zenodo, https://doi.org/10.5281/zenodo.7932319, and from the corresponding author on reasonable request.

## Ethics statement

The studies involving human participants were reviewed and approved by Puerta de Hierro University Hospital Ethics Committee. The patients/participants provided their written informed consent to participate in this study.

## Author contributions

DB participated in the study design, carried out data collection, performed statistical analysis, and drafted the manuscript. AZ carried out data collection and critical review of the manuscript for intellectual content. IM conceived the study and made a critical review of the manuscript for intellectual content. RG-G conceived the study, participated in its design, performed statistical analysis, and revised the manuscript for intellectual content. All authors have read and approved the final manuscript.

## References

[1] Rossini Z Nicolosi F Kolias AG Hutchinson PJ De Sanctis P Servadei F. The history of decompressive craniectomy in traumatic brain injury. Front Neurol. (2019) 10:458. 10.3389/fneur.2019.00458 31133965 PMC6517544

[2] Vahedi K Hofmeijer J Juettler E Vicaut E George B Algra A. Early decompressive surgery in malignant infarction of the middle cerebral artery: a pooled analysis of three randomised controlled trials. Lancet Neurol. (2007) 6:215–22. 10.1016/S1474-4422(07)70036-4 17303527

[3] Danish SF Barone D Lega BC Stein SC. Quality of life after hemicraniectomy for traumatic brain injury in adults. A review of the literature. Neurosurg Focus. (2009) 26:E2. 10.3171/2009.3.FOCUS945 19485715

[4] Cruz-Flores S Berge E Whittle IR. Surgical decompression for cerebral oedema in acute ischaemic stroke. Cochrane Database Syst Rev. (2012) 1:CD003435. 10.1002/14651858.CD003435.pub2 22258954 PMC11491187

[5] Hutchinson PJ Kolias AG Timofeev IS Corteen EA Czosnyka M Timothy J. Trial of decompressive craniectomy for traumatic intracranial hypertension. N Engl J Med. (2016) 375:1119–30. 10.1056/NEJMoa1605215 27602507

[6] van Middelaar T Richard E van der Worp HB van den Munckhof P Nieuwkerk PT Visser MC. Quality of life after surgical decompression for a space-occupying middle cerebral artery infarct: a cohort study. BMC Neurol. (2015) 15:156. 10.1186/s12883-015-0407-0 26311142 PMC4551524

[7] Ling M Zhang Q Zang L Li X Liu Q. Decompressive craniectomy can improve the recovery of neurological function, daily living ability and life quality of patients with intracerebral hemorrhage after surgery. Am J Transl Res. (2021) 13:11364–74. 34786064 PMC8581915

[8] D'Ambrosio AL Sughrue ME Yorgason JG Mocco JD Kreiter KT Mayer SA. Decompressive hemicraniectomy for poor-grade aneurysmal subarachnoid hemorrhage patients with associated intracerebral hemorrhage: clinical outcome and quality of life assessment. Neurosurgery. (2005) 56:12–9. 10.1227/01.NEU.0000144820.38439.63 15617581

[9] Berger N Brunner A Wünsch G Nistl O Pinter D Hofler SF. Long-term outcome after decompressive hemicraniectomy for malignant middle cerebral artery infarction. J Neurol. (2023) 270:1–8. 10.1007/s00415-023-11679-1 37004558 PMC10066964

[10] Benejam B Sahuquillo J Poca MA Frascheri L Solana E Delgado P. Quality of life and neurobehavioral changes in survivors of malignant middle cerebral artery infarction. J Neurol. (2009) 256:1126–33. 10.1007/s00415-009-5083-9 19288045

[11] Vahedi K Benoist L Kurtz A Mateo J Blanquet A Rossignol M. Quality of life after decompressive craniectomy for malignant middle cerebral artery infarction. J Neurol Neurosurg Psychiatry. (2005) 76:1181–2. 10.1136/jnnp.2004.058537 16024906 PMC1739735

[12] Weil AG Rahme R Moumdjian R Bouthillier A Bojanowski MW. Quality of life following hemicraniectomy for malignant MCA territory infarction. Can J Neurol Sci. (2011) 38:434–8. 10.1017/S0317167100011835 21515502

[13] Waqas M Malik N Shamim MS Nathani KR Abbasi SA. Quality of life among patients undergoing decompressive craniectomy for traumatic brain injury using glasgow outcome scale extended and quality of life after brain injury scale. World Neurosurg. (2018) 116:e783–90. 10.1016/j.wneu.2018.05.092 29852303

[14] Rauen K Reichelt L Probst P Schäpers B Müller F Jahn K. Decompressive craniectomy is associated with good quality of life up to 10 years after rehabilitation from traumatic brain injury. Crit Care Med. (2020) 48:1157–64. 10.1097/CCM.0000000000004387 32697486

[15] van Middelaar T Nederkoorn PJ van der Worp HB Stam J Richard E. Quality of life after surgical decompression for space-occupying middle cerebral artery infarction: systematic review. Int J Stroke. (2015) 10:170–6. 10.1111/ijs.12329 25042345

[16] Green TL Newcommon N Demchuk A. Quality of life and caregiver outcomes following decompressive hemicraniectomy for severe stroke: a narrative literature review. Can J Neurosci Nurs. (2010) 32:24–33. 20533642

[17] Kelly ML Shammassian B Roach MJ Thomas C Wagner AK. Craniectomy and craniotomy in traumatic brain injury: a propensity-matched analysis of long-term functional and quality of life outcomes. World Neurosurg. (2018) 118:e974–81. 10.1016/j.wneu.2018.07.124 30048790

[18] Alonso J Prieto L Anto JM. The Spanish version of the SF-36 Health Survey (the SF-36 health questionnaire): an instrument for measuring clinical results. Med Clin (Barc). (1995) 104:771–6. 7783470

[19] Verdugo MÁ Gómez LE Fernández M Aguayo V Arias B. CAVIDACE Quality of Life Scale. Application and Correction Manual. Salamanca: INICO.(2018).

[20] Alonso JM Regidor E Barrio G Prieto L Rodríguez CR Fuente LD. Population reference vaules of the Spanish version of the Health Questionnaire SF-36. Med Clin (Barc). (1998) 111:410–6. 9834913

[21] Broderick JP Adeoye O Elm J. Evolution of the modified rankin scale and its use in future stroke trials. Stroke. (2017) 48:2007–12. 10.1161/STROKEAHA.117.017866 28626052 PMC5552200

[22] Hofmeijer J Kappelle LJ Algra A Amelink GJ van Gijn J van der Worp HB. Surgical decompression for space-occupying cerebral infarction (the Hemicraniectomy After Middle Cerebral Artery infarction with Life-threatening Edema Trial [HAMLET]): a multicentre, open, randomised trial. Lancet Neurol. (2009) 8:326–33. 10.1016/S1474-4422(09)70047-X 19269254

[23] Rahme R Zuccarello M Kleindorfer D Adeoye OM Ringer AJ. Decompressive hemicraniectomy for malignant middle cerebral artery territory infarction: is life worth living? J Neurosurg. (2012) 117:749–54. 10.3171/2012.6.JNS111140 22920962

[24] Jüttler E Unterberg A Woitzik J Bösel J Amiri H Sakowitz OW. Hemicraniectomy in older patients with extensive middle-cerebral-artery stroke. N Engl J Med. (2014) 370:1091–100. 10.1056/NEJMoa1311367 24645942

[25] Medeiros GC Roy D Kontos N Beach SR. Post-stroke depression: a 2020 updated review. Gen Hosp Psychiatry. (2020) 66:70–80. 10.1016/j.genhosppsych.2020.06.011 32717644

[26] Curry WT Jr Sethi MK Ogilvy CS Carter BS. Factors associated with outcome after hemicraniectomy for large middle cerebral artery territory infarction. Neurosurgery. (2005) 56:681–92. 10.1227/01.NEU.0000156604.41886.62 15792506

[27] Foerch C Lang JM Krause J Raabe A Sitzer M Seifert V. Functional impairment, disability, and quality of life outcome after decompressive hemicraniectomy in malignant middle cerebral artery infarction. J Neurosurg. (2004) 101:248–54. 10.3171/jns.2004.101.2.0248 15309915

[28] Woertgen C Erban P Rothoerl RD Bein T Horn M Brawanski A. Quality of life after decompressive craniectomy in patients suffering from supratentorial brain ischemia. Acta Neurochir (Wien). (2004) 146:691–5. 10.1007/s00701-004-0280-x 15197612

[29] Sundseth J Sundseth A Thommessen B. Long-term outcome and quality of life after craniectomy in speech-dominant swollen middle cerebral artery infarction. Neurocrit Care. (2015) 22:6–14. 10.1007/s12028-014-0056-y 25127905

